# Research hotspots and trends related to pain in gouty arthritis from 2014 to 2024: A bibliometric analysis

**DOI:** 10.1097/MD.0000000000040525

**Published:** 2024-11-15

**Authors:** Chengyin Lu, Yuxing Guo, Zhiqiang Luo, Xiaomei Hu, Hui Xiong, Yang Xiang, Yang Shu, Gonghui Jian

**Affiliations:** a The Second Clinic College of Traditional Chinese Medicine, Hunan University of Chinese Medicine, Changsha, China; b Department of Orthopedics, The Affiliated Hospital of Hunan Academy of Traditional Chinese Medicine, Changsha, China; c Department of Orthopedics, The First Hospital of Hunan University Chinese Medicine, Changsha, China; d Department of Orthopedics, Hunan Provincial People’s Hospital (The First Hospital of Hunan Normal University), Changsha, China; e College of Integrative Chinese and Western Medicine, Hunan University of Chinese Medicine, Changsha, China.

**Keywords:** bibliometric analysis, CiteSpace, gouty arthritis, pain, VOSviewer

## Abstract

**Background:**

Gouty arthritis is a metabolic condition caused by disordered purine metabolism and elevated uric acid levels. This study adopts a bibliometric approach to analyze current research on pain in gouty arthritis and forecast future research trends.

**Methods:**

Retrieve relevant research on gouty arthritis pain in the Web of Science core collection database, screen literature, and use visualization software such as CiteSpace, Vosviewers, and R package “Bibliometrix” for analysis.

**Results:**

The total number of documents included was 1133, with 909 articles and 224 reviews. Between 2014 and 2024, there was an overall upward trend in the number of publications about pain in gouty arthritis, with the United States of America and China ranking first and second, respectively, in terms of publication volume. The UNIVERSITY OF ALABAMA BIRMINGHAM had the most publications, and Professor DALBETH N played a key role in this field. According to the keyword analysis, disease management and treatment, particularly methods for enhancing patients’ quality of life and reducing symptoms, are research hotspots. For a deeper understanding, attention is also being paid to the epidemiology and pathological mechanisms of the disease. Emerging keywords such as “gut microbiota” and “urate-lowering therapies” indicate growing interest in the interrelationship between gut microbiota and gout, and the development of new treatment methods.

**Conclusion:**

This bibliometric study reveals that research on gouty arthritis pain is actively developing. Current hot topics reflect investigations into the deeper pathological mechanisms of gouty arthritis and the development of new treatment methods, including urate-lowering therapies. There is also increasing attention on the role of gut microbiota in the disease. Despite limitations such as the preliminary nature of research methods and insufficient interdisciplinary collaboration, future research directions aim to improve the rigor of research design, strengthen international cooperation, promote unified treatment guidelines, and optimize the diagnosis and treatment of gouty arthritis with new technologies like artificial intelligence, precision medicine, and nanomedicine. This will drive the field towards a deeper scientific understanding, more effective treatment methods, and more comprehensive disease management, ultimately improving patients’ prognosis and quality of life.

## 1. Introduction

Gouty arthritis (GA) is a metabolic condition caused by disordered purine metabolism and elevated uric acid levels, leading to monosodium urate (MSU) crystal deposition around joints, triggering local inflammation and tissue destruction.^[1–3]^ The acute flares of GA are characterized by rapid-onset joint pain, redness, swelling, and heat, with pain intensifying within 6 to 12 hours.^[4]^ These flares often affect the first metatarsophalangeal joint, severely impacting patients’ quality of life.^[5]^ With the global incidence of GA rising, particularly among younger populations, effective pain management is crucial.^[6]^

In recent years, advances in understanding the pathophysiology of gout have led to significant progress in treatment, particularly in pain management during acute flares.^[7]^ Current main treatments include medication, cold treatment, and physical therapy.^[8–10]^ Medications primarily comprise nonsteroidal antiinflammatory drugs, NSAIDs (such as etoricoxib), corticosteroids (such as prednisone), and specific gout medications (such as colchicine and allopurinol), which work by inhibiting purine synthesis, reducing the production and deposition of uric acid, and thereby alleviating pain and inflammation.^[11]^ Cold treatment and physical therapy, such as soft tissue techniques and joint mobilization, can also help relieve joint inflammation and pain.^[12]^ Additionally, complementary therapies like traditional Chinese medicine, acupuncture, or herbal treatments have provided some relief for gout patients’ pain.^[4,13–15]^ However, challenges remain, such as adverse drug reactions, disease recurrence, and patient compliance, impacting long-term outcomes and quality of life.^[16]^ Despite growing research, bibliometric analyses summarizing advancements in gout pain treatment remain limited, with a need for comprehensive assessments to understand current trends and future directions.

Bibliometrics, as a tool capable of evaluating research trends and hotspots through quantitative analysis, offers a unique perspective for a comprehensive understanding of the research landscape in the field of gout pain treatment.^[17]^ Therefore, in order to gather and visualize extensive data on nations, authors, institutions, journals, keywords, and research areas related to pain in GA, we used sophisticated bibliometric tools in this study, such as CiteSpace,^[18]^ VOSviewer,^[19]^ R package “Bibliometrix.”^[20]^ This helps us to better understand pain in GA and provides data and direction for future research priorities by outlining the research hotspots, development trends, and future directions in the field.

## 2. Methods

### 2.1. Search strategy

**Figure 1.**
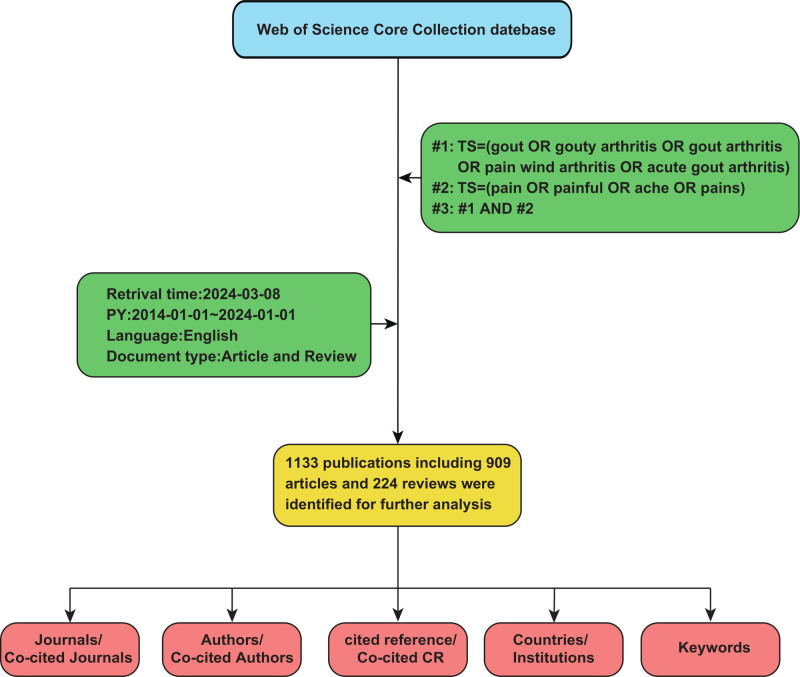
Flowchart of literature identification and analysis process. CR = cited reference, PY = Year Published, TS = Topic, WOSCC = Web of Science core collection.

A thorough search of the literature was carried out in the WOSSC database, taking into account that the database’s daily updates could affect the studies included. Using the following search formula, the literature search was finished within one day (March 8, 2024):#1: TS = (gouty arthritis) OR TS = (gout arthritis) OR TS = (acute gout arthritis) OR TS = (pain wind arthritis) OR TS = (gout), #2: TS = (pain) OR TS = (painful) OR TS = (ache) OR TS = (pains), finally =#1 AND#2. Language was restricted to English, and the types of literature were limited to articles and reviews. The publication period in this study was set from January 1, 2014, to January 1, 2024. Following the initial search, the literature is screened based on inclusion and exclusion criteria. The inclusion criteria for the literature were as follows: studies with complete bibliographic information; studies related to gouty arthritis pain. The exclusion criteria included: duplicate publications, studies with incomplete bibliographic information that prevented data retrieval, studies unrelated to gouty arthritis pain, and non-research materials such as conference notices, letters, or news reports. The retrieved literature was first manually screened by reviewing titles and keywords, then deduplicated using Zotero (Version 6.0.36), resulting in the final selection of included studies. Figure 1 depicts the flowchart for the screening procedure. For this bibliometric analysis, ethical approval was not necessary as it did not involve direct human or animal subjects, relying solely on public data and literature.

### 2.2. Data analysis

Following data collection, we retrieved significant bibliometric information from the selected publications, such as publication volumes by year, countries, regions, publishing institutions, journals, authors, citation data, and keywords, among others. To quantify and illustrate the above data, 4 programs were used for analysis: VOSviewer (Version 1.6.18), the R package “bibliometrix,” CiteSpace (Version 6.3.R1), and an online analytic platform (https://bibliometric.com).

## 3. Results

### 3.1. Annual trends in publications

**Figure 2.**
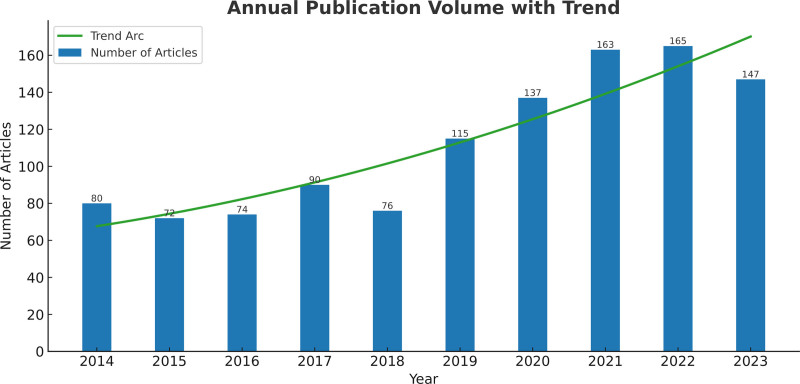
Annual output of pain in gouty arthritis.

Using the methodology outlined in Figure 1, we gathered 1133 relevant publications in all, consisting of 909 articles and 224 reviews. Figure 2 shows the annual number of publications, growing from 80 in 2014 to 147 in 2023, with a trend arc indicating a generally rising trend in publication volume. From 2014 to 2018, there were no significant shifts in research related to gouty arthritis pain. However, from 2019 onwards, research in this area began to increase annually, rising from 115 publications in 2019 to 165 in 2022. On one hand, this increase is attributed to the rising incidence of GA due to global aging, changes in lifestyle, and other various factors, which has garnered more attention from the public and researchers, thus driving the increase in related research. On the other hand, it also relates to the continuous advancement in biomedical technologies in recent years, such as developments in molecular biology, bioinformatics, and genomics, providing researchers with more tools and resources to delve into the mechanisms and treatment methods for pain in GA. In summary, the overall trend of annual publication volume indicates that publications related to pain in GA are continuously increasing, demonstrating that research activities in the field of GA are active and likely to continue growing in the coming years.

### 3.2. Distribution of countries/regions

**Figure 3.**
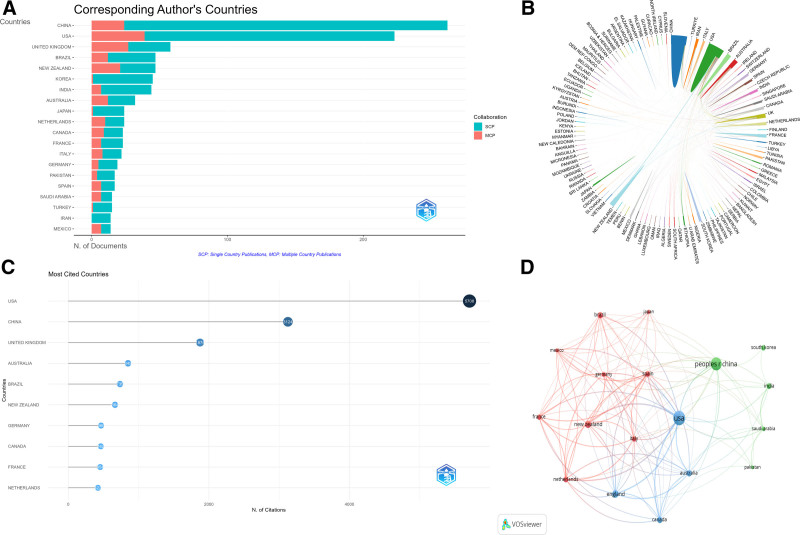
Country distribution of pain in gouty arthritis. (A) Top 20 countries that produced the largest number of literature; (B) The international cooperation analysis among different countries; (C) Top 10 most cited countries; (D) The overlay visualization map of country co-authorship analysis. MCP = Multiple Country Publications, SCP = Single Country Publications.

The publication volume of countries/regions can be estimated based on the “Corresponding Author’s Countries,” as shown in Figure 3A, among all 1133 publications, they originated from 73 countries and regions. The two countries with the highest number of publications were China (N = 262, 23.12%) and the United States of America (N = 223, 19.68%). Both countries had more than 200 publications, which is significantly more than those of other countries or regions. The United Kingdom of Great Britain and Northern Ireland (UK) came next (N = 58, 0.05%), with publications also exceeding 50. In terms of cooperation between countries/regions, as shown in Figure 3A, although China ranks first in the number of publications, it has less collaboration with other countries/regions, whereas USA and UK have richer academic exchanges with other countries/regions in the field of pain in GA. From Figure 3B and the country cooperation network in Table 1, it is shown that USA has coauthored more than 20 publications with the UK, New Zealand, Canada, Spain, and the Netherlands. Additionally, as can be seen from Figure 3C’s ranking of the nations with the most citations, the United States of America unquestionably leads in terms of citations, with 5708, considerably above China’s 3124, demonstrating the country’s dominance in the field of pain in GA.

**Table 1:** Top 10 countries in terms of cooperation quantity

Rank	From	To	Frequency
1	USA	UNITED KINGDOM	26
2	USA	NEW ZEALAND	25
3	USA	CANADA	23
4	USA	SPAIN	21
5	USA	NETHERLANDS	20
6	UNITED KINGDOM	NEW ZEALAND	18
7	UNITED KINGDOM	SPAIN	16
8	USA	AUSTRALIA	16
9	USA	CHINA	16
10	NETHERLANDS	ITALY	14

Furthermore, Figure 3D shows that the collaboration between countries/regions is divided into three clusters, with the countries in these clusters mostly from Europe, the Americas, and Asia, indicating that international cooperation in this field has certain geopolitical characteristics.

### 3.3. Institutional analysis

**Figure 4.**
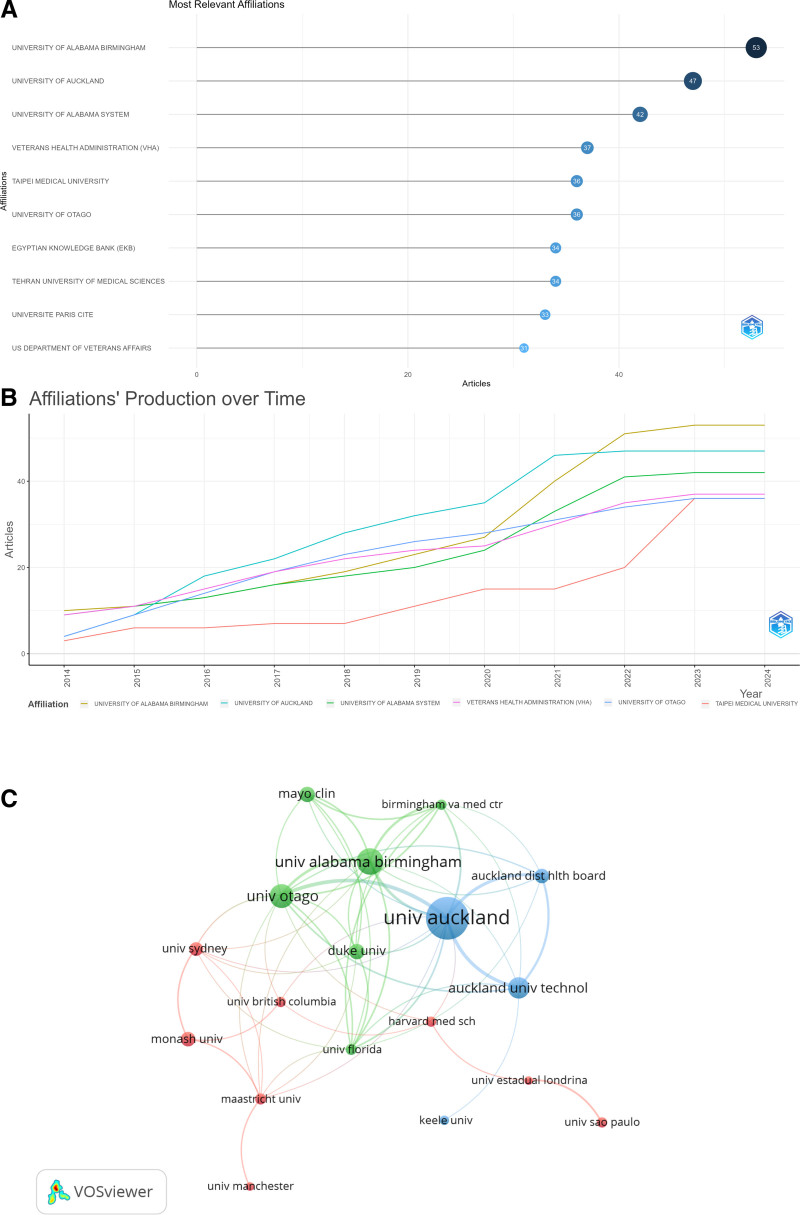
Institutional status of pain in gouty arthritis publications. (A) Top 10 institutions by the number of publications; (B) Top 5 institutions production over time (TAIPEI MEDICAL UNIVERSITY and UNIVERSITY OF OTAGO are tied for fifth place); (C) The visulization of institutions cooperation networks.

As shown in Figure 4A, the UNIVERSITY OF ALABAMA BIRMINGHAM leads the Top 10 institutions in terms of publications with 53 articles, followed by the University of Auburn (47 articles). Each of the Top 10 institutions has published over 30 articles. The annual publication counts of the Top 5 publishing institutions are shown in Figure 4B, indicating a steady annual increase in publications for all five institutions (with TAIPEI MEDICAL UNIVERSITY and UNIVERSITY OF OTAGO each publishing 36 articles, tied for fifth). The network of institutions with more than 10 publications, as shown in Figure 4C, divides the publishing institutions for publications related to GA pain into three clusters, with the collaboration hubs centered around the UNIVERSITY OF AUCKLAND, UNIVERSITY OF ALABAMA BIRMINGHAM, and UNIVERSITY OF SYDNEY, suggesting that these three institutions are the core institutions in the field of GA pain research.

### 3.4. Analysis of journals

**Figure 5.**
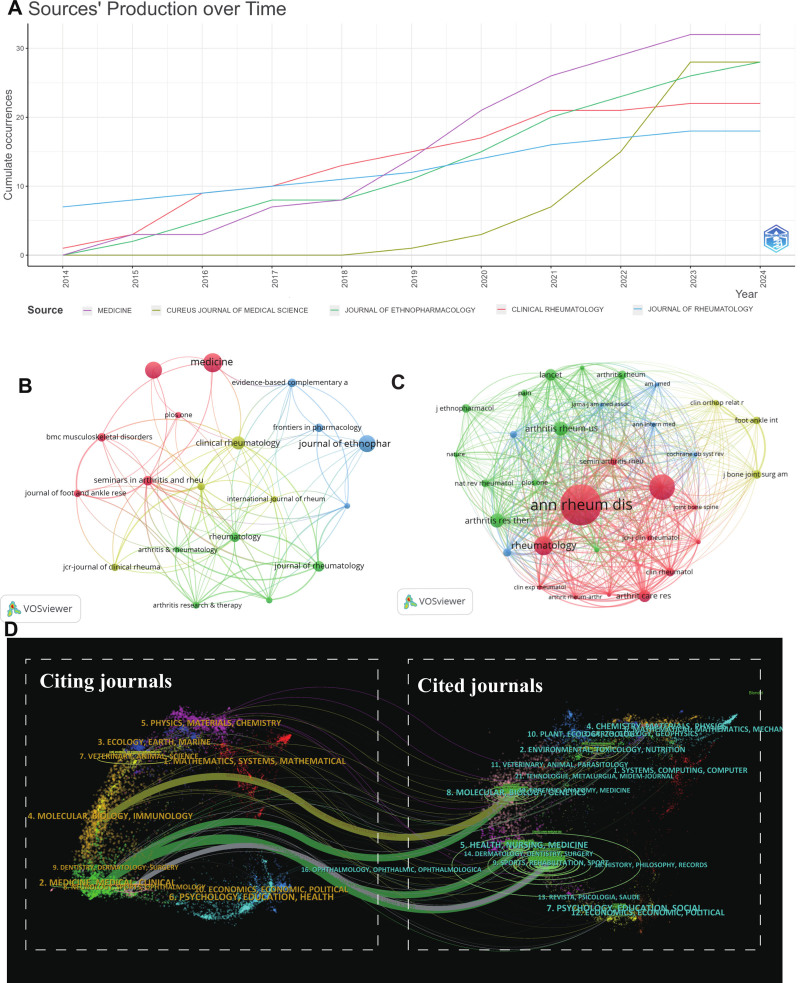
Journal status of pain in gouty arthritis publications. (A) Top 5 journals production over time; (B) Network map of journals that were cited in more than 10 citations; (C) Network map of journals that were co-cited in more than 200 citations; (D) The dual-map overlay of journals related to pain in gouty arthritis.

The research on pain associated with GA is published across 508 sources. Figure 5 and Table 2 show the situation of journals publishing on pain in GA. Table 2 shows the Top 10 journals with the highest publication volume, with the Top three being MEDICINE (N = 32), CUREUS JOURNAL OF MEDICAL SCIENCE (N = 28), and J ETHNOPHARMACOL (N = 28). However, the ranking in terms of journal citation counts significantly differs from the publication volume, with ANN RHEUM DIS (N = 2041) being the highest, followed by J RHEUMATOL (N = 1271) and RHEUMATOLOGY (N = 959). Among them, the journal with the highest impact factor is LANCET (IF = 168.9), ranked Q1. Figure 5A reveals the growth curves over time for the Top 5 journals by volume of publications. Overall, since 2014, the volume of publications related to pain in GA has been increasing annually for each journal. Figure 5B shows the collaboration network of journals that have published more than 10 articles on pain in GA, featuring 20 journals centered around four nodes: MEDICINE, J RHEUMATOL, J ETHNOPHARMACOL, and CLIN RHEUMATOL, mainly divided into four clusters, closely collaborating with other journals. Figure 5C displays the collaboration network of journals cited over 200 times, with 31 journals cited over 100 times divided into four clusters, with ANN RHEUM DIS in a central position.

**Table 2:** Top 10 journals and citations journals of gouty arthritis pain research

Rank	Journals	Articles	IF	Q	Rank	Journals	Citetions	IF	Q
1	MEDICINE	32	1.6	3	1	ANN RHEUM DIS	2041	27.4	1
2	CUREUS J MED SCIENCE	28	1.2	/	2	J RHEUMATOL	1271	3.9	2
3	J ETHNOPHARMACOL	28	5.4	2	3	RHEUMATOLOGY	959	5.5	1
4	CLIN RHEUMATOL	22	3.4	3	4	ARTHRITIS RES THER	714	4.9	2
5	J RHEUMATOL	18	3.9	2	5	ARTHRITIS RHEUM-US	701	/	/
6	FOOT ANKLE INT	17	2.7	2	6	ARTHRIT CARE RES	615	4.7	2
7	RHEUMATOLOGY	16	5.5	1	7	LANCET	576	168.9	1
8	SEMIN ARTHRITIS RHEU	16	2	2	8	J ETHNOPHARMACOL	437	5.4	1
9	BMC MUSCULOSKEL DIS	14	2.3	2	9	J BONE JOINT SURG AM	435	5.3	1
10	EVID-BASED COMPL ALT	14	/	/	10	ARTHRITIS RHEUM	429	/	/

Dual-Overlays is one of the unique applications of CiteSpace software, visualizing research areas and citation trajectory by simultaneously displaying citing and cited maps. As shown in Figure 5D, the clusters on the left represent journals citing the work, while those on the right represent journals that are cited. The main pathway shows that articles published in the fields of Molecular/Biology/Genetics, Health/Nursing/Medicine, and Sports/Rehabilitation/Sport are primarily cited by researchers in the journals of Molecular/Biology/Immunology and Medicine/Medical/Clinical. The results of the journal’s dual-map overlays may indicate that research on pain in GA exhibits strong interdisciplinary characteristics, mainly focusing on analytical biology, genetics, nursing, and sports rehabilitation, with these areas’ findings receiving significant attention from researchers in molecular biology, immunology, and clinical medicine. This may reflect the research hotspots in GA pain focusing on unraveling pathophysiological mechanisms and developing new diagnostic and treatment methods.

### 3.5. Analysis of authors

**Figure 6.**
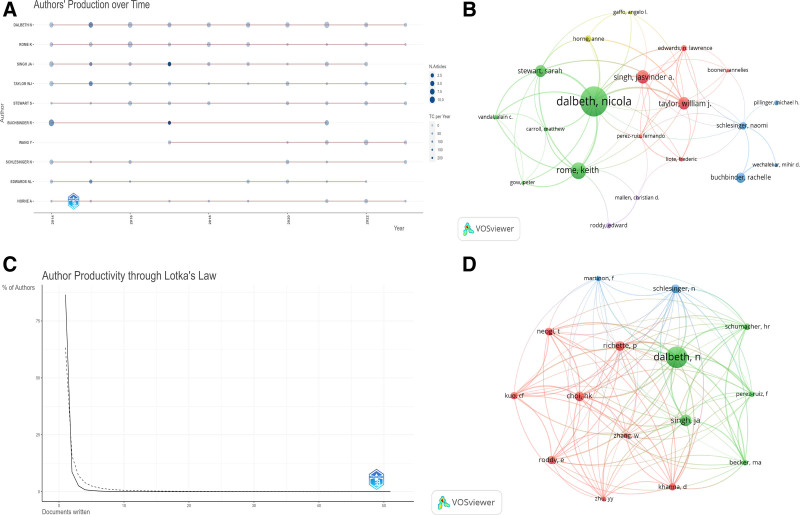
Author status of pain in gouty arthritis publications. (A)Top 10 authors’ production over time (B) Network map of authors that were cited in more than 6 citations; (C) Author productivity through Lotka’s Law; (D) Network map of authors that were co-cited in more than 100 citations.

A total of 6188 authors have participated in research on pain in GA. Figure 6 and Table 3 depict the author’s status in terms of GA pain. The Top 10 authors have published a total of 212 publications, representing approximately 18.7% of all papers published in this subject. The most prolific author is DALBETH N from the University of Auckland, New Zealand, with 51 papers, followed by ROME K from AUT University, New Zealand (N = 28), and SINGH JA from the University of Alabama at Birmingham, USA (N = 25). In the list of the Top 10 most-cited authors, the Top three are DALBETH N (New Zealand), TAYLOR WJ (New Zealand), and SINGH JA (USA), with DALBETH N leading with 296 citations. As shown in Table 3, DALBETH N has the highest H-index at 16. DALBETH N is not only the author with the most publications and citations but also holds the highest h-index, indicating his core position in this field. Figure 6A displays a timeline for the Top 10 authors by publication volume, with nine of the Top 10 authors having started their research on pain in GA in 2014 and continuing to do so. Lotka’s Law describes the relationship between the productivity of authors and the number of articles in scientific production. According to Lotka’s Law, a few authors in a field will produce a large number of articles, while the majority will produce only a few. Figure 6C shows that the productivity and publication number distribution of authors on GA pain roughly align with the expected outcomes of Lotka’s Law.

**Table 3:** Top 10 authors and citetions authors of gouty arthritis pain research

Rank	Authors	Articles	H-index	Country	Institutions	Rank	Authors	Citations	Country	Institutions
1	DALBETH N	51	16	New Zealand	University of Auckland	1	DALBETH N	296	New Zealand	University of Auckland
2	ROME K	28	11	New Zealand	AUT University	2	TAYLOR WJ	208	New Zealand	University of Otago
3	SINGH JA	25	13	USA	University of Alabama	3	SINGH JA	194	USA	University of Alabama
4	TAYLOR WJ	22	12	New Zealand	University of Otago	4	SCHUMACHER HR	134	USA	University of Pennsylvania
5	STEWART S	19	9	New Zealand	University of Auckland	5	NEOGI T	133	USA	Boston University School of Medicine
6	BUCHBINDER R	18	14	Australia	Monash University	6	PEREZ-RUIZ F	133	Spain	Hospital Universitario Cruces
7	WANG Y	14	5	China	Peking University First Hospital	7	EDWARDS NL	121	Germany	Technical University Dresden
8	SCHLESINGER N	13	8	USA	University of Utah	8	TAUSCHE AK	114	UK	University of Edinburgh
9	EDWARDS NL	11	7	USA	University of Florida	9	ROME K	107	New Zealand	AUT University
10	HORNE A	11	6	UK	University of Brighton	10	NUKI G	106	UK	University of Edinburgh

Figure 6B represents the collaboration relationship among authors whose published article has been cited 6 times on pain in GA, totaling 33 authors and mainly divided into 5 clusters, with DALBETH N (New Zealand), HORNE A (UK), SCHLESINGER N (USA), TAYLOR WJ (New Zealand), and ROODDY E (UK) serving as focal points. Figure 6D shows the collaborative network of authors who have been co-cited more than 100 times, with a total of 15 authors divided into three clusters, centered around DALBETH N (New Zealand), CHOI HK (USA), and SCHLESINGER N (USA). Interestingly, authors from New Zealand play a significant role in this field, which may be due to New Zealand being one of the countries with the highest incidence of gout, especially among the Maori and Pacific Islander populations who have specific genetic backgrounds that lead to a very high incidence of gout. This has stimulated researchers’ interest in studying pain in GA and provided rich data and cases for clinical and epidemiological research, thus placing New Zealand authors in an important position in this field.

### 3.6. Highly cited publications and references bursts

Table 4 shows the top 10 most cited publications and references. The most cited publication is “KUO CF, 2015, NAT REV RHEUMATOL,^[21]^” with 73 citations. This publication provides a review of the global epidemiology of gout: prevalence, incidence, and risk factors. The article indicates that the distribution of gout globally is uneven, with the highest prevalence in Pacific countries. Developed countries tend to have a higher burden of gout compared to developing countries, and there seems to be an increasing trend in both prevalence and incidence of the disease. Certain ethnic groups are particularly susceptible to gout, underscoring the importance of genetic predisposition. The second-ranked article is “NEOGI T, 2015, ARTHRITIS RHEUMATOL,^[22]^” which represents the 2015 gout classification criteria published in collaboration by the American College of Rheumatology and the European League Against Rheumatism. Developed through data-driven and decision analysis approaches, this classification standard features outstanding performance characteristics and incorporates the latest evidence on gout. It has been widely recognized and used around the world. The most cited reference is the 2006 article published in Nature, “Gout-associated uric acid crystals activate the NALP3 inflammasome.^[23]^” This study reveals how MSU triggers the activation of the NALP3 inflammasome by caspase-1 during the pathogenesis of gout, leading to the production of active interleukin (IL)-1β and IL-18. This provides a crucial molecular basis for understanding the inflammatory mechanism of gout pain and lays the groundwork for developing new therapeutic strategies against gout, aiding in the discovery of new drug targets or treatment methods.

**Table 4:** Top 10 cited publications and references of gouty arthritis pain research

Rank	Document	Citations	Rank	Cited references	Citations
1	KUO CF, 2015, NAT REV RHEUMATOL	71	1	MARTINON F, 2006, NATURE, V440, P237	94
2	NEOGI T, 2015, ARTHRITIS RHEUMATOL	42	2	WALLACE SL, 1977, ARTHRITIS RHEUM, V20, P895	92
3	NEOGI T, 2015, ANN RHEUM DIS	33	3	ZHU YY, 2011, ARTHRITIS RHEUM-US, V63	88
4	QASEEM A, 2017, ANN INTERN MED-a	23	4	RICHETTE P, 2017, ANN RHEUM DIS, V76, P29	77
5	CHANDRATRE P, 2013, RHEUMATOLOGY	22	5	KHANNA D, 2012, ARTHRIT CARE RES, V64, P1431	76
6	TOPROVER M, 2015, CURR RHEUMATOL REP	22	6	KHANNA D, 2012, ARTHRIT CARE RES, V64, P1447	73
7	RICHETTE P, 2020, ANN RHEUM DIS	20	7	KUO CF, 2015, NAT REV RHEUMATOL, V11, P649	71
8	TERKELTAUB RA, 2013, ARTHRITIS RES THER	17	8	DALBETH N, 2016, LANCET, V388, P2039	65
9	HAINER BL, 2014, AM FAM PHYSICIAN	16	9	RICHETTE P, 2010, LANCET, V375, P318	65
10	HOFFMEISTER C, 2014, RHEUMATOLOGY	11	10	ZHANG W, 2006, ANN RHEUM DIS, V65, P1312	106

**Figure 7.**
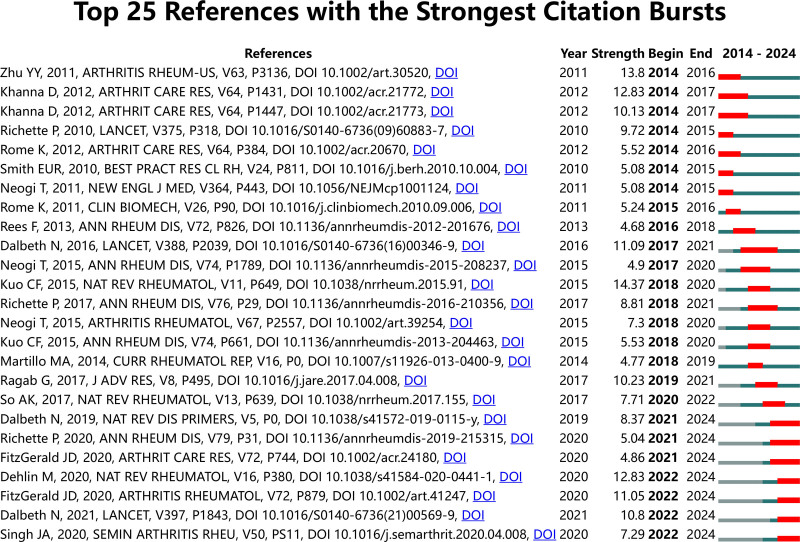
Top 25 references with the Strongest Citation Bursts.

References widely cited within a certain timeframe on a given topic are termed as having citation bursts. These are significant metrics that underscore references stimulating interest within a specific area during a defined period. Figure 7 shows the top 25 references with the strongest citation burst. Consistent with the most cited document, “KUO CF, 2015, NAT REV RHEUMATOL^[21]^” ranks highest (strength = 14.37). It is followed by “ZHU YY, 2011, ARTHRITIS RHEUM-US^[24]^” (strength = 13.8), which estimated the prevalence of gout and hyperuricemia based on a nationally representative sample of US men and women (National Health and Nutrition Examination Survey [NHANES] 2007–2008) and suggested that the prevalence of gout and hyperuricemia may be associated with increased frequencies of obesity and hypertension. The longest sustained citation burst was for “Dalbeth N, 2016, LANCET, V388, P2039,^[25]^” a review that comprehensively summarized the pathogenesis, diagnosis, and treatment of GA, widely cited continuously from 2017 to 2021.

### 3.7. Keywords analysis

#### 3.7.1. Keywords with strongest citation bursts

**Figure 8.**
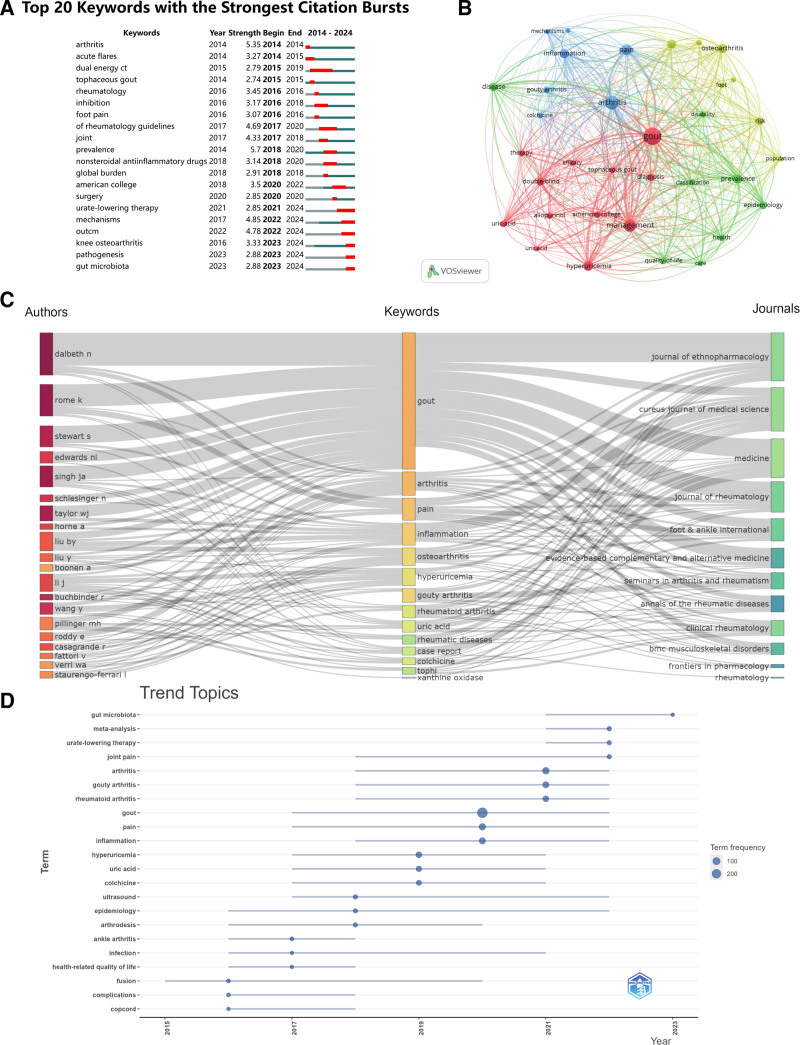
Keywords status of wrist arthroscopy publications. (A) Top 20 Keywords with the strongest citation bursts; (B) Keyword network map with keywords appearing more than 30 times; (C) Three-field plot of the Keywords analysis; (D) The historical migration of research hotspots.

By analyzing keyword bursts, one can acquire a better knowledge of research topics of interest throughout a certain time period and determine the direction of research progress. The Top 20 Keywords with the Strongest Citation Bursts are shown in Figure 8A. The keyword “prevalence” leads with the highest strength (5.7), showing a burst period from 2018 to 2020, followed by “arthritis” (strength = 5.35) bursting in 2014, and “mechanisms” (strength = 4.85) from 2022 to 2024. The term “urate-lowering therapy” has the longest burst period in recent years, lasting from 2021 to 2024 for 3 years. This suggests that research in the area of GA pain has been focusing on understanding the disease’s prevalence, investigating treatment approaches, and delving into its underlying mechanisms, indicating a shift from broad epidemiological studies to detailed mechanism investigations. This trio of keywords’ collective analysis reveals a trend towards future research prioritizing disease prevention, diagnosis, and treatment – particularly through in-depth mechanism understanding to innovate new treatments. The continued burst of “urate-lowering therapy” in recent years signifies an active exploration of the fundamental cause of gout pain – hyperuricemia. This focused study on strategies to lower uric acid levels marks a research shift from merely managing gout symptoms to tackling the cause itself. Through the explosive growth of this keyword, we can see that related research is not only aiming to alleviate the short-term suffering of gout patients but, more importantly, is delving into and understanding the fundamental cause of gout pain – the abnormal elevation of uric acid levels.

#### 3.7.2. Keyword co-occurrence analysis

We use VOSviewer for co-occurrence analysis of keywords in this field. As shown in Figure 8B, Out of a total of 2715 keywords, there are 34 keywords that appear more than 30 times, divided into four clusters corresponding to red, yellow, green, and blue. The red cluster’s keywords primarily focus on the treatment, diagnosis, and management of gout and hyperuricemia; the yellow cluster includes keywords related to long-term complications associated with gout and overall health conditions of patients, pointing to the impact of GA on the feet and chronic disease risks; the green cluster concentrates on the epidemiology, classification, and patients’ quality of life regarding gout; the blue cluster’s theme centers on the core issues of arthritis, particularly pain and inflammation related to GA, as well as the exploration of pathological mechanisms. Based on the frequency of keywords, the Top 5 keywords are “management,” “arthritis,” “gout,” “prevalence,” and “rheumatoid-arthritis” (Table 5). From the ranking of keyword frequencies, it can be observed that research hotspots in the field of pain in GA primarily focus on the disease’s management and treatment. This particularly includes strategies to improve patients’ quality of life and alleviate symptoms, while also paying attention to the epidemiology and pathological mechanisms of the disease for a more in-depth understanding. Moreover, research on pain management and hyperuricemia are key themes, as they directly impact disease management and patients’ quality of life.

**Table 5:** Top 20 keywords of gouty arthritis pain research

Rank	Keywords	Occurrences	Centrality
1	management	170	0.02
2	arthritis	154	0.06
3	gout	94	0.07
4	prevalence	94	0.1
5	rheumatoid-arthritis	91	0.05
6	disease	78	0.07
7	pain	76	0.01
8	risk	72	0.08
9	hyperuricemia	70	0.08
10	double-blind	69	0.05
11	diagnosis	59	0.05
12	epidemiology	59	0.06
13	inflammation	57	0.1
14	osteoarthritis	57	0.12
15	uric-acid	54	0.11
16	American-college	51	0.03
17	quality-of-life	49	0.05
18	health	44	0.06
19	therapy	43	0.01
20	care	39	0.06

#### 3.7.3. Three-field plot analysis

To better comprehend the associations between authors, keywords, and publishing journals, Figure 8C displays a three-field plot of this data. The most frequently used keywords are “gout,” “arthritis,” “pain,” “inflammation,” and “osteoarthritis.” Authors DALBETH N, ROME K, and STEWART S have established the strongest connections with the keywords “gout” and “pain.” At the same time, the strongest links are most closely associated to the journals J ETHNOPHARMACOL, CUREUS JOURNAL OF MEDICAL SCIENCE, and MEDICINE, showing that these journals have published the bulk of publications containing the aforementioned keywords.

#### 3.7.4. Keyword timeline analysis

By analyzing the most frequently occurring keywords in the field of gouty arthritis pain at different stages, we can understand the research focus during this period and predict future research directions. As shown in Figure 8D, from 2014 to 2024, a total of 22 keywords appeared. Over the last decade, research on pain in GA has experienced several pivotal changes. Initially, the emphasis was placed on diagnosing and treating the condition, reflecting the researchers’ focus on alleviating acute symptoms and enhancing the precision of diagnoses. Subsequently, the research focus shifted towards the epidemiology of gout and long-term disease management strategies, with an increased emphasis on studies related to urate-lowering therapy, reflecting a focus on sustained and preventative treatments. In recent years, the frequency of the keyword “gut microbiota” has risen, indicating a shift in research focus towards emerging areas related to gout. Researchers have begun to explore the connection between gut microbiota and gout, considering how this new discovery might impact the treatment and management of gout. Overall, from the provided timeline of keywords, we can observe the continuous evolution of research in the field of pain in GA, showcasing a trajectory from macro to micro, and then to a holistic approach. For future research in GA pain, the expected trend will likely focus more on the interrelationship between gut microbiota and gout, as well as further research and application of urate-lowering therapies.

## 4. Discussion

### 4.1. Progress in GA pain research

GA is a metabolic joint disorder caused by high uric acid levels, leading to the crystallization of monosodium urate salts and their deposition around joints, triggering inflammatory responses and tissue damage.^[26]^

Acute flares present with joint redness, swelling, heat, and pain, particularly affecting the first metatarsophalangeal joint, but also the knees, ankles, and wrists.^[27]^ Recent research has revealed the Uric acid plays a pivotal role in GA pain, with hyperuricemia as its pathological basis influenced by genetics and environmental factors. MSU crystals, when detached by stimuli like injury or cold, enter the joint space and activate immune cells, triggering inflammatory signaling pathways that induce GA.^[28,29]^ Building on this, the NLRP3-centered inflammatory cascade can directly regulate the occurrence of pain. The NLRP3 inflammasome, upon activation, enhances pain by releasing pro-inflammatory cytokines IL-1β and IL-18, which contribute to pain.^[30]^ The “inflammation-pain” amplification effect is also an important manifestation of “pathological presentation-clinical symptoms” in the process of GA.

Currently, the primary treatments for pain in GA involve NSAIDs, corticosteroids, and colchicine to quickly alleviate inflammation and pain during acute flares.^[31–33]^ However, these drugs have notable side effects. Although NSAIDs are widely used, prolonged or excessive use can lead to stomach ulcers, gastric bleeding, gastrointestinal perforation, kidney damage, and an increased risk of heart attacks and strokes.^[34]^ Corticosteroids, while potent anti-inflammatory agents, can elevate fracture risk, suppress immune responses (increasing infection risk), complicate blood sugar control in diabetics, and cause mood instability, weight gain, and edema with long-term use.^[35]^ Colchicine, a classic gout treatment, can induce gastrointestinal issues like nausea and diarrhea, affect bone marrow leading to anemia, and cause liver and kidney toxicity, alongside potential drug interactions.^[36,37]^ Furthermore, these medications only address inflammation and pain without lowering uric acid levels, the root cause of GA, resulting in condition recurrence. Consequently, patients often require urate-lowering drugs like febuxostat and probenecid. Therefore, current clinical treatments for GA pain have significant limitations.

In recent years, research into GA-related pain has deepened, leading to an increase in related publications. However, despite the numerous studies in this field, our understanding of overall research trends, hotspots, and international collaboration remains limited. By analyzing the development trends and research hotspots of GA pain over the past decade, we can gain insights into the field’s evolution, identify research opportunities, and anticipate potential future challenges. This approach not only facilitates a better evaluation of developments in GA pain but also provides valuable guidance and inspiration for future research. Therefore, this study employed literature visualization methods to analyze publications related to GA pain in the WOSSCC database, revealing key research hotspots and trends.

### 4.2. Analysis of results

By analyzing the results of this study, we can identify research hotspots related to pain in GA and predict future trends. First, global research on GA pain has intensified, with a significant increase in publications since 2019. This rise is linked to the worsening global aging situation and lifestyle changes (such as dietary habits and lack of exercise), leading to higher incidence rates of gout and arthritis.^[6]^ Additionally, the rapid advancement of fields like molecular biology, bioinformatics, and genomics has provided new tools and perspectives for studying the pathological mechanisms, diagnostic methods, and treatment approaches for GA pain.^[38,39]^ urthermore, the growing public health awareness and pursuit of improved quality of life have motivated researchers to explore more effective management strategies to mitigate the disease’s impact on patients’ lives. Thus, current trends suggest that research activities on pain in GA are expected to continue to grow in the coming years.

In terms of institutions for GA pain, the University of Auckland and the University of Alabama are in a significant leading position in this field. As a top research institution in New Zealand, the University of Auckland possesses leading scholars in the field of GA, such as Professor DALBETH N and Professor STEWART S. Additionally, the university possesses outstanding research teams and facilities in areas such as clinical medicine, biomedical science, and life sciences, offering strong academic and resource support for gout research. In terms of inter-institutional cooperation, these institutions actively collaborate, forming a closely connected international cooperation network.^[40,41]^ For example, these institutions jointly drafted the American College of Rheumatology Guidelines for the Management of Gout.^[42]^ They also jointly conduct multicenter clinical trials, providing new ideas and methods for the treatment of GA.^[43]^

According to the author, DALBETH N holds a prominent place in this subject, being both the most cited and the most published author. His high H-index signifies his leadership in GA pain research. DALBETH N is a highly reputed expert in the field of rheumatology. Especially, he possesses deep insights and rich research experience in gout and other diseases related to uric acid. He currently serves as a professor at the University of Auckland, New Zealand, and as a senior consultant in rheumatology at the University of Auckland’s Faculty of Medical and Health Sciences. His research involves basic science experiments, innovative applications of imaging techniques, and the design and execution of large clinical trials.^[44–46]^ He is the principal investigator of several significant studies, including those on urate transporters and various pharmacological treatment strategies.^[47]^ Additionally, he has investigated the impact of gout on patients’ quality of life and its association with cardiovascular diseases.^[48]^

Through keyword analysis, we can see an increased focus on epidemiology, exploration of treatment methods, and a deeper understanding of pathological mechanisms in the field of pain in GA. The burst of the keyword “prevalence” signifies the increasing concern of public health experts and clinicians towards gout and its impact on public health. With lifestyle changes, particularly dietary habits, the global prevalence of gout is on the rise, prompting researchers to investigate its epidemiological characteristics to develop effective prevention and management strategies. The prominence of the keyword “mechanisms” indicates that scientists are delving into the molecular and cellular mechanisms of gout onset, aiding in understanding its fundamental causes and providing a theoretical basis for new treatment targets and strategies. The recent surge in “urate-lowering therapy” reflects the urgent need for fundamental treatment methods for gout. The focus of this therapy is not only on controlling acute symptoms but also on preventing worsening and recurrence by lowering blood uric acid levels.^[49,50]^ This reveals a research trend shifting from symptomatic treatment to fundamental disease treatment. Keyword analysis reveals that research on pain in GA has shifted from singular treatment methods to comprehensive disease management, covering multiple aspects including prevention, early diagnosis, treatment, and patient education. This shift reflects the need for all-encompassing care for patients with gout, emphasizing the importance of personalized treatment plans and lifestyle interventions. Meanwhile, the emergence of “gut microbiota” suggests that research will increasingly incorporate microbiomics, exploring the relationship between gout and the human microbiome.^[51,52]^ This interdisciplinary trend broadens the understanding of gout and may uncover new treatment methods.

### 4.3. Limitations and predictions

Based on the analysis above, we believe that research in the field of GA pain still has the following limitations: Although research on pain in GA has a wide international impact, cooperation between different countries and research institutions is still not sufficient. Currently, some research methods within the field of GA pain, including the establishment of animal models and exploration of clinical treatment methods, may still be in preliminary or observational stages. For instance, due to the presence of uricase in rats, simply elevating uric acid levels in rats cannot simulate the human gout onset mechanism. Instead, it requires the injection of sodium urate crystals into rat joints to simulate arthritis inflammation. This also leads to the need for more empirical research to verify and refine the effectiveness, safety, and optimal application methods of these new approaches. Although existing research extensively covers the treatment and management of GA, research on causes, early prevention, and the impact of the disease on patients’ quality of life is still relatively lacking, necessitating further research. Based on this, we believe the future research trends in GA pain have the following directions: Verifying the efficacy and safety of treatment methods through rigorous research approaches such as large-scale, multicenter randomized controlled trials, while also strengthening basic research to deeply understand the pathological mechanisms of GA. Encouraging and promoting cooperation between countries and research institutions, jointly conducting research projects, sharing research findings and technological innovations. Making full use of new technologies such as gene editing, nanomedicine, and targeted drug delivery, and methods like biomarkers and liquid biopsy, to achieve breakthroughs in the prevention, early diagnosis, and treatment options for GA.

In summary, the future trends in research on pain in GA suggest that research will evolve towards a deeper understanding of the disease, comprehensive management, personalized treatment, and interdisciplinary exploration. These trends not only guide future research directions but also provide new ideas and strategies for clinical practice.

## 5. Conclusion

In this bibliometric analysis, we examined the current state of research in the field of GA pain. From 2014 to 2024, the number of papers related to pain in GA overall showed an upward trend. The United States and China are the two countries with the highest number of publications. The UNIVERSITY OF ALABAMA BIRMINGHAM emerges as the institution with the most publications. Professor DALBETH N occupies a central position within this domain. Additionally, keyword analysis reveals the hotspots and future directions of research, including terms like “prevalence,” “mechanisms,” and “urate-lowering therapy,” “gut microbiota,” indicating that the focus of research has gradually deepened over time. These efforts will push the field of GA pain towards a deeper scientific understanding, more effective treatment methods, and more comprehensive disease management approaches, thus improving patient prognosis and quality of life.

## Author contributions

**Data curation:** Chengyin Lu, Zhiqiang Luo, Xiaomei Hu, Yang Shu, Gonghui Jian.

**Software:** Zhiqiang Luo, Xiaomei Hu, Yang Xiang, Yang Shu, Gonghui Jian.

**Visualization:** Zhiqiang Luo, Xiaomei Hu, Yang Xiang, Yang Shu, Gonghui Jian.

**Writing – original draft:** Chengyin Lu, Yuxing Guo, Hui Xiong.

**Writing – review & editing:** Chengyin Lu, Yuxing Guo, Hui Xiong.
